# Gene4HL: An Integrated Genetic Database for Hearing Loss

**DOI:** 10.3389/fgene.2021.773009

**Published:** 2021-10-18

**Authors:** Shasha Huang, Guihu Zhao, Jie Wu, Kuokuo Li, Qiuquan Wang, Ying Fu, Honglei Zhang, Qingling Bi, Xiaohong Li, Weiqian Wang, Chang Guo, Dejun Zhang, Lihua Wu, Xiaoge Li, Huiyan Xu, Mingyu Han, Xin Wang, Chen Lei, Xiaofang Qiu, Yang Li, Jinchen Li, Pu Dai, Yongyi Yuan

**Affiliations:** ^1^ College of Otolaryngology Head and Neck Surgery, Chinese PLA General Hospital, Chinese PLA Medical School, Beijing, China; ^2^ National Clinical Research Center for Otolaryngologic Diseases, State Key Lab of Hearing Science, Ministry of Education, Beijing, China; ^3^ Beijing Key Lab of Hearing Impairment Prevention and Treatment, Beijing, China; ^4^ National Clinical Research Center for Geriatric Disorders, Department of Geriatrics, Xiangya Hospital, Central South University, Changsha, China; ^5^ Center for Medical Genetics & Hunan Key Laboratory, School of Life Sciences, Central South University, Changsha, China; ^6^ Angen Gene Medicine Technology Co., Ltd., Beijing, China

**Keywords:** hearing loss, database, gene4HL, genetic variants, genotype, phenotype

## Abstract

Hearing loss (HL) is one of the most common disabilities in the world. In industrialized countries, HL occurs in 1–2/1,000 newborns, and approximately 60% of HL is caused by genetic factors. Next generation sequencing (NGS) has been widely used to identify many candidate genes and variants in patients with HL, but the data are scattered in multitudinous studies. It is a challenge for scientists, clinicians, and biologists to easily obtain and analyze HL genes and variant data from these studies. Thus, we developed a one-stop database of HL-related genes and variants, Gene4HL (http://www.genemed.tech/gene4hl/), making it easy to catalog, search, browse and analyze the genetic data. Gene4HL integrates the detailed genetic and clinical data of 326 HL-related genes from 1,608 published studies, along with 62 popular genetic data sources to provide comprehensive knowledge of candidate genes and variants associated with HL. Additionally, Gene4HL supports the users to analyze their own genetic engineering network data, performs comprehensive annotation, and prioritizes candidate genes and variations using custom parameters. Thus, Gene4HL can help users explain the function of HL genes and the clinical significance of variants by correlating the genotypes and phenotypes in humans.

## Introduction

Hearing loss (HL) is one of the most common disabilities, affecting 1.57 billion people globally in 2019, accounting for 20% of the global population (Collaborators 2021). In industrialized countries, out of every 1,000 newborns, 2-3 have HL. Various factors can result in HL, including genetic factors, environmental factors, and unknown factors. Among them, about 60% of HL is caused by genetic factors, including 70% non-syndromic hearing loss (NSHL) and 30% syndromic hearing loss (SHL) (Morton et al., 2006). Hereditary HL is a typically monogenic disorder and is highly heterogeneous. Until now, 170 NSHL loci have been identified, involving 124 related genes (http://hereditaryhearingloss.org/, 2021-08) (Van et al., 2021), and more than 400 syndromes associated with HL have been described (Marazita et al., 1993). With the development of next-generation sequencing (NGS), targeted gene capture and sequencing panel, whole-exome sequencing (WES), and whole-genome sequencing (WGS) have been used in HL diagnosis, resulting in the identification of more and more candidate gene variants in patients with HL (Cabanillas et al., 2018).

Plenty of studies have researched the pathogenicity of the candidate genes and genetic variants as well as their related phenotypes, but the results are scattered in different literatures. Up to March 31, 2021, a total of 16,354 studies could be retrieved from PubMed using “genetic” and “hearing loss” as the keywords. Previous studies have usually collected detailed information on the genotype-phenotype correlations of HL genes and variants, mainly through literature retrieval. However, this technique is not only complicated but can also result in loss of important information, further resulting in an inaccurate judgment of the pathogenicity of gene variants as well as an incomplete understanding of their related phenotypes. Therefore, there is an urgent need to develop an integrative database of candidate genes and genetic variants in HL.

Currently, there are many valuable, free, public databases on hereditary diseases, which can be used for various tasks ranging from simple data-finding to more authentic retrieval and analysis. Of them, only the following two databases have been specifically designed for HL: Hereditary Hearing Loss Homepage (https://hereditaryhearingloss.org) (Van et al., 2021) and Deafness Variation Database (DVD, http://deafnessvariationdatabase.org/) (Hela et al., 2018). Hereditary Hearing Loss Homepage mainly provides users with information about loci, deafness genes, and related literatures but does not include specific variant information. DVD focuses on the variant classification based on collected evidence and is curated by experts in genetic HL to provide a single-source guide to variant interpretation. Although DVD is highly accepted and widely used worldwide, it does not include comprehensive phenotypes of variants. In addition, the ClinVar database (https://www.ncbi.nlm.nih.gov/clinvar/) (Landrum et al., 2016) is also one of the databases commonly used by researchers working with hereditary diseases, which integrates data regarding genetic variations and clinical phenotypes. However, the ClinVar database relies on voluntary submission from various researchers and institutions. An analysis of submissions to the ClinVar variant database of the National Center for Biotechnology Information (NCBI) (Landrum et al., 2014) revealed that the interpretation of the importance of the same variant by multiple clinical laboratories might differ; thus, at least one interpretation must be wrong, which could lead to inappropriate medical intervention (Rehm et al., 2015), risking the integrity of information. Therefore, it is necessary to conduct a thorough collection, systematic integration, and detailed annotation of all candidate genes and variants involved in the onset and development of HL from public databases.

In this study, we developed a one-stop database of HL-related genes and variants, Gene4HL, to facilitate the understanding of the genetic basis of HL. We systematically searched and manually reviewed the literature in PubMed, followed by cataloging almost all HL-related genes and variants. Combined with molecular epidemiological data sources, the function of each gene and related variations annotation were integrated into the database. Furthermore, we characterized the spatiotemporal expression pattern and functional network of HL-related genes to facilitate the understanding of the pathophysiology of deafness. Thus, Gene4HL provides a comprehensive genetic understanding of HL by creating an analytic platform for researchers and clinicians.

## Material and Methods

### Data Collection

**FIGURE 1:**
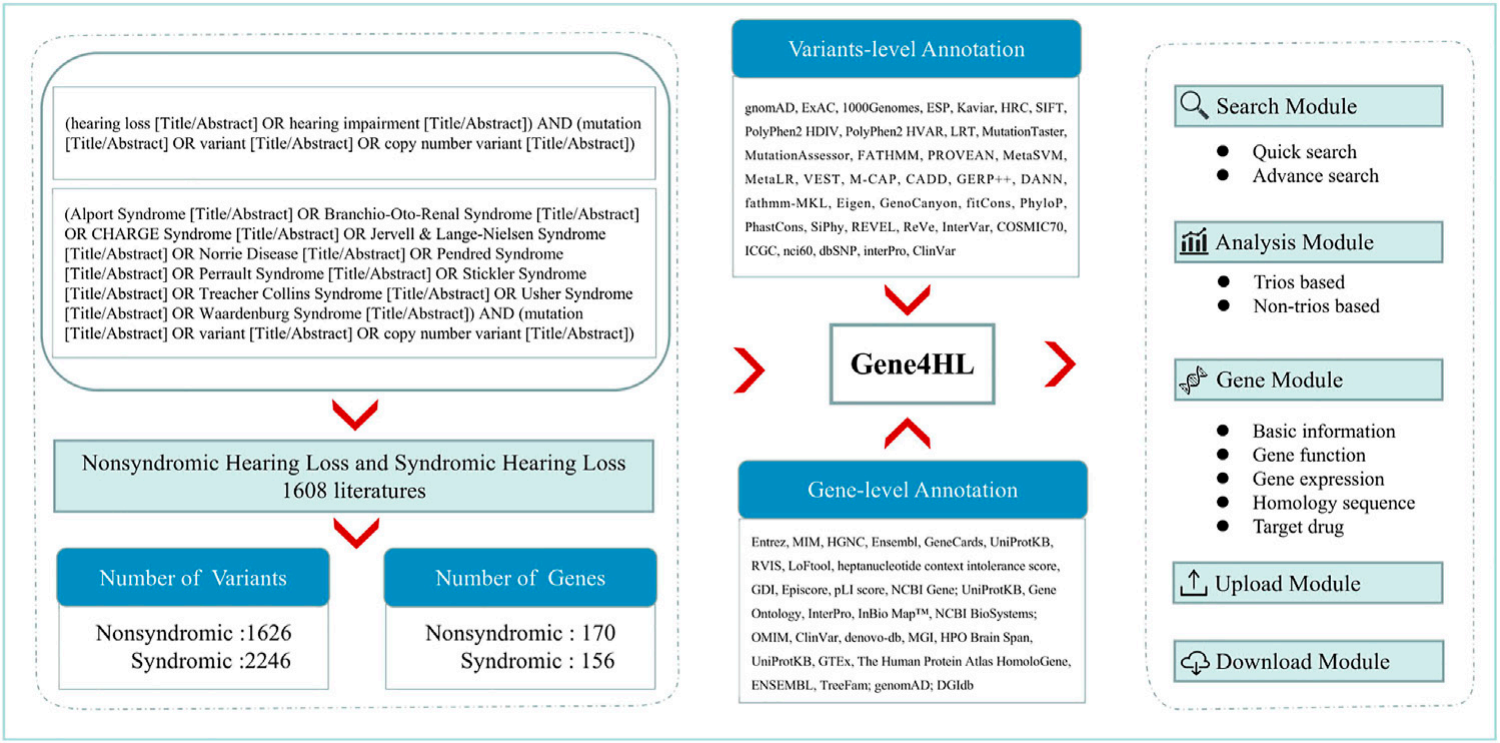
The overall roadmap of this study. The left section shows the process of data collection and analysis of Gene4HL. Gene4HL supports search module, analysis module, gene module, upload, and download module, as shown in the right section. The middle section shows the annotation information at the variant level and gene level.

We retrieved the relevant publications in the PubMed database up to March 31, 2021, to obtain the complete and detailed information of genes and variants related to HL. The keywords used for searching were “(hearing loss OR hearing impairment) AND (mutation OR variant) (Title/Abstract).” For SHL, common diseases with HL, including Alport Syndrome (Kandai et al., 2019; Nozu et al., 2019), Branchio-Oto-Renal Syndrome (Kochhar et al., 2007), CHARGE Syndrome (Hsu et al., 2014), Jervell & Lange-Nielsen Syndrome (Cusimano et al., 1991), Norrie Disease (Sowden et al., 2020), Pendred Syndrome (Reardon et al., 1996), Perrault Syndrome (Pan et al., 2020), Stickler Syndrome (David 2002), Treacher Collins Syndrome (Dixon 1996), Usher Syndrome (Mathur et al., 2019), Waardenburg Syndrome (Pingault et al., 2010) were searched according to Hereditary Hearing Loss Homepage (Van et al., 2021). Next, all HL-related publications were reviewed manually. The annotation of genes and variants, detailed information of clinical data of patients (ethnicity, hearing, and other symptoms) were extracted through in-depth reading of the full text of each publication (Figure 1).

### Comprehensive Annotation

Gene4HL integrated the information of 62 public databases (Supplementary Table S1) and annotated six aspects from the gene level. The meaningful annotations for each gene were collected from public databases, including basic information, phenotype and disease, protein sequences, protein-protein interaction, gene expression pattern, and gene functions, drug-gene interactions, and the gene-drug abilities for precision medicine. Each extracted variation was annotated in detail using ANNOVAR (Wang et al., 2010), such as the functional effects and functional prediction of variants as well as allele frequencies of variants in different populations based on the definitions of transcripts from RefSeq. The annotations of each variation included the basic information (location, PubMed ID, variant type, genetic pattern, the allele frequencies of different populations, etc.), predictive scores and pathogenicity, variant and related diseases or phenotype information, the total genetic testing samples, the positive samples, etc.

### Gene Prioritization

Based on the functional effects and ReVe (Rare Exome and Variant Effect Scoring Tool) score, which were previously proposed by our group (Li et al., 2018), variants were divided into five classes: 1) loss-of-function (LoF) variants, including stop-gain, stop-loss and splicing variants, 2) damaging missense variants (ReVe score >0.7), 3) tolerant missense variants (ReVe score ≤0.7), 4) synonymous variants, 5) non-frameshift indels variants, including non-frameshift deletion, non-frameshift insertion, non-frameshift substitution, and 6) noncoding variants.

Next, we extended our previously developed scoring system in other genetic diseases (Li et al., 2021; Zhao et al., 2021) to prioritize genes associated with HL to quantify the contribution of the different classes of variants and integrate them into the entire prioritization of deafness genes (Supplementary Table S2). Briefly, the LoF variants were assigned an evidence score of five; the damaging missense variants and tolerant missense variants were assigned an evidence score of 3 and 2, respectively; the non-frameshift indels and noncoding variants were assigned an evidence score of 1. An integrated summing up evidence score for each gene was calculated by adding the evidence scores of each type of variant from all integrated studies. All genes integrated in this study were classified into three classes: high confidence (score ≥20), strongly associated (score of 10–20), and suggestively associated (score of 5–10). We performed a permutation test to evaluate the interconnectivity and functional correlation among the HL-related genes (score ≥5). Specifically, we randomly simulated 1,000,000 permutation tests to evaluate the interconnectivity among the HL-related genes.

### Spatiotemporal Expression Pattern Analysis

The spatiotemporal transcriptase of the mouse inner-ear organs (cochlea and utricle) at four developmental stages (E16, P0, P4, and P7) were sourced from a previous study (Shen et al., 2015) (https://shield.hms.harvard.edu/). We applied signed hybrid-weighted gene co-expression network analysis (WGCNA) (Langfelder et al., 2008) to analyze all 16 samples using the standard method with a power of eight to cluster the spatiotemporal-expression patterns and prenatal laminar-expression profiles of a given gene set.

### Functional Network Analysis

A permutation test was performed to investigate the interconnectivity and functional correlation of HL-related genes (evidence score ≥5) using the STRING v11.0 database (Szklarczyk et al., 2019). In addition, we constructed a protein-protein interaction network using the STRING online analysis platform (https://string-db.org/) with a confidence score >0.4. Moreover, the functional networks were clustered by multiple biological processes of Gene Ontology (GO) (The Gene Ontology 2017) (http://www.geneontology.org/).

### Developing of Gene4HL Database

Gene4HL was developed, supported by versatile browsing and searching functionalities, similar to the Gene4Denovo database (Zhao et al., 2020). All data were stored in a MySQL database. Users could access the genetic data or extended analysis results freely through this web interface. The web interface of Gene4HL contained Search, Analysis, Browse, Upload, and Download modules.

## Results

### Data and Database Overview

A total of 1,608 publications met the inclusion criteria and were used for extracting further information. Gene4HL database (http://www.genemed.tech/gene4hl/) integrated 326 HL-related genes, including 170 NSHL-related genes and 156 SHL-related genes, involving 3,872 genetic variations. The corresponding detailed genetic and clinical information was also integrated into the Gene4HL database. The database was comprehensively annotated at the variant-level and gene-level, including 1) functional effects of variants (nonsense, nonsynonymous, and frameshift, etc.), 2) disease and phenotype-related information for variant- and gene-level implications, 3) functional consequences of variants through 24 in silico predictive algorithms, 4) allele frequency in different populations of public databases, 5) meaningful gene-level information, such as protein sequences, protein-protein interactions, the gene expression patterns in human tissues, and gene functions, etc., and 6) drug-gene interactions and the gene-drug abilities for precision medicine (Figure 1).

Notably, we found that 7 of the 10 most frequently occurring variants of Gene4HL were located on *GJB2*, the most common HL gene associated with DFNB1A and DFNA3A (Supplementary Figure S1A). Next, we classified variants based on the DVD and the American College of Medical Genetics and Genomics (ACMG) guidelines for the interpretation of variants in the context of Gene4HL. The variants were classified as pathogenic (P), likely pathogenic (LP), variant of uncertain significance (VUS), likely benign (LB), or benign (B). Of these variants, 2,115 (54.7%) were classified as P, 748 (19.3%) were LP, 127 (3.3%) were LB, 474 (12.2%) were B, and 406 (10.5%) were VUS (Supplementary Figure S1B). Of the 3,873 variants listed in the Gene4HL, 2,343 were found in the DVD.

### Quick and Advance Searching in Gene4HL

**FIGURE 2:**
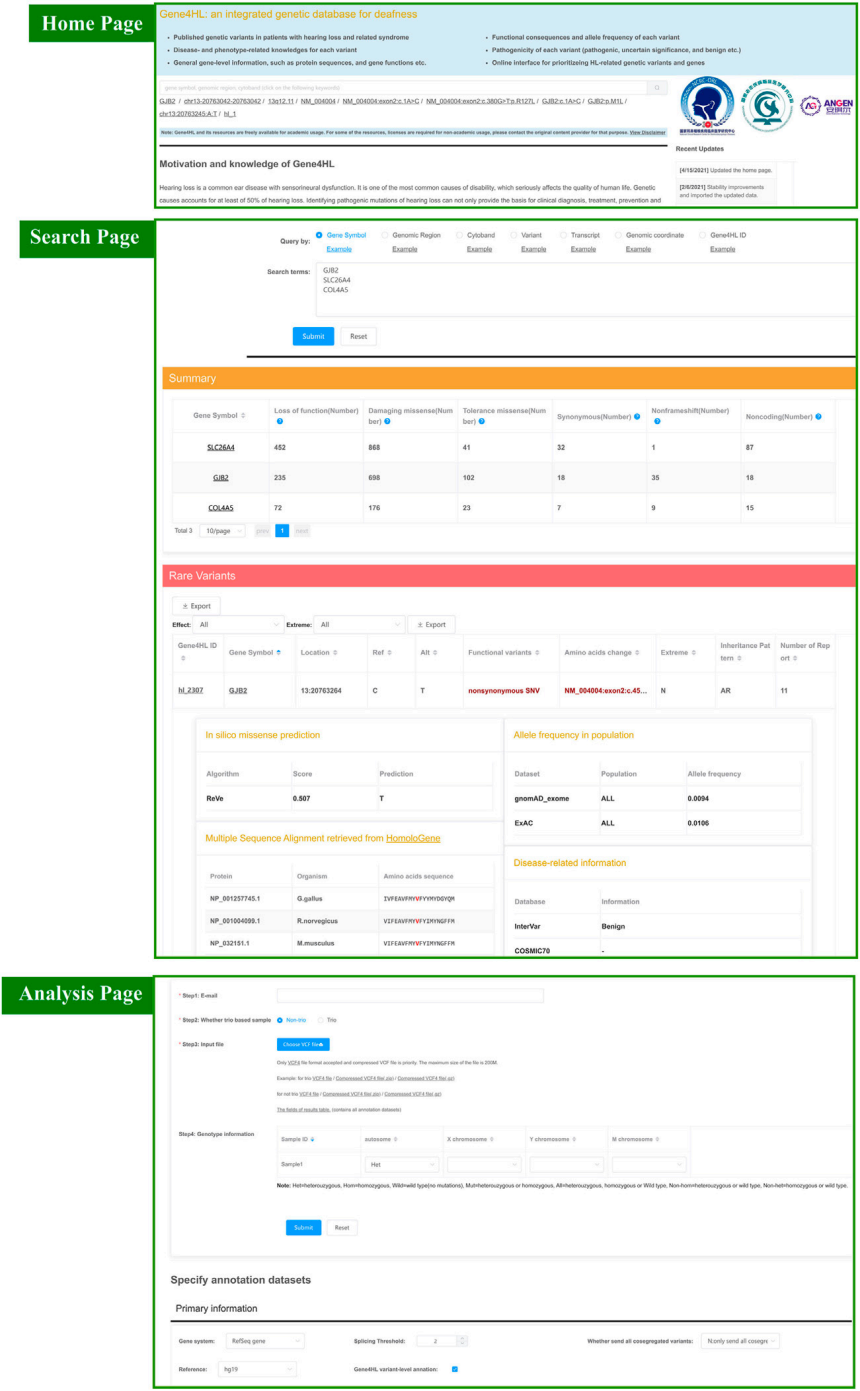
Snapshot of search module and analysis module in Gene4HL. The top section shows a snapshot of the quick search module and home page in Gene4HL. The middle section shows a snapshot of the search module in Gene4HL. The bottom section shows a snapshot of the analysis module in Gene4HL.

The query interface contains panels for quick and advanced searching. The quick searching function is the main tool for quick access to detailed information regarding genes or variations, which can be found on the homepage. The quick search automatically identifies seven key terms, such as gene symbol, genomic region, cytoband, transcript accession, the nucleic acid change in a certain gene or transcript, the genomic coordinate of a variant, and the Gene4HL ID (Figure 2).

Advanced searching supports batch search and allows users to specify annotated datasets (Figure 2). The advanced searching provides options for primary information, prediction algorithms for nonsynonymous variations, allele frequencies in different populations, and disease-related and phenotype-related information. The advanced searching also has the same seven input forms like quick searching. The advanced searching query form and the corresponding result set have been displayed on the same page to improve the users’ experience. Also, users can specify datasets and obtain various information.

Both quick and advanced searching results provide detailed annotation data of genes and variations. The searching results are contained in two tables, a summary of the variation of each gene and detailed annotation at the variant level. The summary table presents the number of loss of function, damaging missense, tolerance missense, synonymous, non-frameshift, and noncoding variants. The detailed table contains the specific information of each variant: the Gene4HL ID, gene symbol, location, functional variant, amino acids change, the pathogenicity, inheritance pattern, and a number of reports. In this searching result interface, users can access annotation on gene-level by clicking the gene symbol in the table. Gene4HL integrates the information of the main public databases and annotates six aspects from the gene level: 1) Basic information, including the official name, location, gene type, gene summary (UniProt 2018), the genic intolerance score (Forge et al., 2003; Itan et al., 2015; Petrovski et al., 2015; Aggarwala et al., 2016; Lek et al., 2016; Fadista et al., 2017; Han et al., 2018); 2) Gene function, including the molecular function, gene ontology terms (The Gene Ontology 2017), domain information (Finn et al., 2017), protein–protein interactions (Li et al., 2017), and biological pathway information (Geer et al., 2010); 3) Phenotype and disease, including phenotype data (Amberger et al., 2015), clinical variation data (Landrum et al., 2016), mammalian phenotype (Eppig et al., 2017), and human phenotype ontology (Kohler et al., 2017); 4) Gene expression, including spatio-temporal expression profiles (Miller et al., 2014), gene expression data in tissues (GTEx Consortium, et al., 2017), and the subcellular location (Uhlen et al., 2015); 5) Variants in different populations; 6) Drug–gene interactions (Wagner et al., 2016).

### Custom-Analysis in Gene4HL

Gene4HL provides users with the interface to freely analyze their own genetic data. In this section, users can upload data of genetic variants (VCF4 format) to identify the co-segregated variants associated with HL and Gene4HL, prioritizing the HL-risk genes in this panel. This upload process involves four simple steps: 1) enter your e-mail address, 2) choose options Trio or Non-trio, 3) upload your file, and 4) fill up trio information or genotype information. Then, datasets of your interest need to be specified and submitted. The main annotation data sets that can be selected are: primary information (gene system, splicing threshold, whether to send all co-segregated variants, reference and Gene4HL variant-level annotation), identify co-segregated variants, perform comprehensive annotations (basic information annotation, pathogenicity prediction of missense variants, allele frequency in various populations, related clinical databases), rare damaging variants (in silico predictive algorithms for nonsynonymous variants, allele frequency in different populations). Concurrently, users can specify their cut-off values of quality control, the annotation data sources, and the methods to detect rare damaging variants. The results are sent to the users’ e-mail once the annotation completes.

Gene4HL also contains other five useful sections: 1) Browse, to facilitate users to browse the HL-related genes in Gene4HL; 2) Upload, to encourage the users to upload their variants list to Gene4HL database; 3) Download, to allow users to freely access all released datasets in Gene4HL and export and download as Excel files; 4) Data Source included in Gene4HL; 5) Tutorial, which provides a further description of Gene4HL.

### HL-Related Genes Prioritization

We developed a weighted scoring system to prioritize HL-related genes by combining all the above genetic evidence. We prioritized 71 high confidence genes (score ≥20), 42 strong associated genes (20 > score ≥10), 52 suggestive associated genes (10 > score ≥5), total of 164 genes (Table 1).

**TABLE 1:** Prioritized candidate genes in Gene4HL.

High confidence genes (score≥20)	Strong candidate genes (20 > score ≥10)	Suggestive candidate genes (10 > score ≥5)
*ABL1, ACTG1#,ADGRV1#, ALMS1, ANKRD11, ANOS1, ATP6V1B1, CDC14A#, CDH23#, CHD7#, CIB2#, CLDN14#, COCH#,COL2A1#, COL4A3#, COL4A4#, COL4A5#, COL11A1#, COL11A2#, EDNRB#,EDN3#, ESPN#, EYA1#, EYA4#, GIPC3#,GJB2#, GPSM2#, KARS#, KCNJ10#, KCNH2, KCNQ1#, KCNQ4#, LHFPL5#, LOXHD1#, MARVELD2#, MYH9#, MYH14#, MITF#, MYO7A#, MYO15A#, MSL3, MYO6#, NDP#, OTOF#, OTOGL#, PAX3#, PCDH15#, PDZD7#, PJVK#, POLR1D#, POU3F4#, POU4F3#, PTPN11, PTPRQ#, SIX1#, SLC4A11,, SLC19A2, SLC26A4#, SMPX#, SOX10#, TBC1D24#,TCOF1#, TECTA#, TMC1#, TMPRSS3#, TRIOBP#, USH1C#, USH1G#, USH2A#, WFS1#*	*ABHD12, ACVR1, ASNS, ATP1A3, BSND#,CABP2#, CEP78, CHD4, CRYM#, DIAPH1#, ESRRB#, FGFR3, FRAS1, GRXCR1#, HARS2#, ILDR1#, KCNE1#, KCNE1B, LOC654841, MAN2B1,MYO3A#, NOG, OTOA#, OTOG#, PEX26, POLG, RMND1, RDX#, SCN5A, SERPINB6#, SLC12A2#, SLC29A3, SOST, STRC#, TBL1X, TJP2#,TMIE#, TXNL4A, TWNK#, WHRN#,XYLT2, ZBTB20*	*ABCC1, AIFM1#,ALG11, BDP1#, BTD, CD164#,CEACAM16#, COL1A1, CREBBP, DNAJC19, DSPP, EFTUD2, ELMOD3#, ENPP1, ERCC8, FAM136A,FGF3, FLNB, GJB4,GJB6#, GJC3, GRHL2#, GRID2,LAMA5, LMX1A#, LRTOMT#, LZTR1, MET#, MYO1A, NDRG1, NEFL, NEUROG1, NLRP3#, OPA1, PLS1#, PRPH2, PRPS1#, PAX2, PCARE, PNPLA2, P2RX2#, RIPOR2#, RP1L1, RS1, RSPO1,SH3TC2, SLC17A8#, SLC26A5#, SLC52A2, SLC52A3, SUCLG1, SYNE4#*

# 98 known HL-causing genes (Van et al., 2021, 60 known HL-causing genes in 70 high confidence genes, 20 known HL-causing genes in 42 strong associated genes, 18 known HL-causing genes in 52 suggestive associated genes). 164 HL-associated genes (score ≥ 5) were classified into three classes, including 70 high confidence genes (score ≥ 20), 42 strong associated genes (score of 10-20), and 52 suggestive associated genes (score of 5–10).

Furthermore, we performed a permutation test based on random resampling, which was used to assess whether functional interconnections of 164 HL-related genes were more than random expectation (evidence score ≥5, Table 1). The permutation test took gene length into consideration. We observed 135 of 164 HL-related genes (*p* < 10^–6^) that interacted with each other and had 809 interconnections (*p* < 10^–6^), which were significantly higher than the random expectation (Supplementary Figure S2).

### Expression Patterns of HL-Related Genes Involved in Inner Ear Development

**FIGURE 3:**
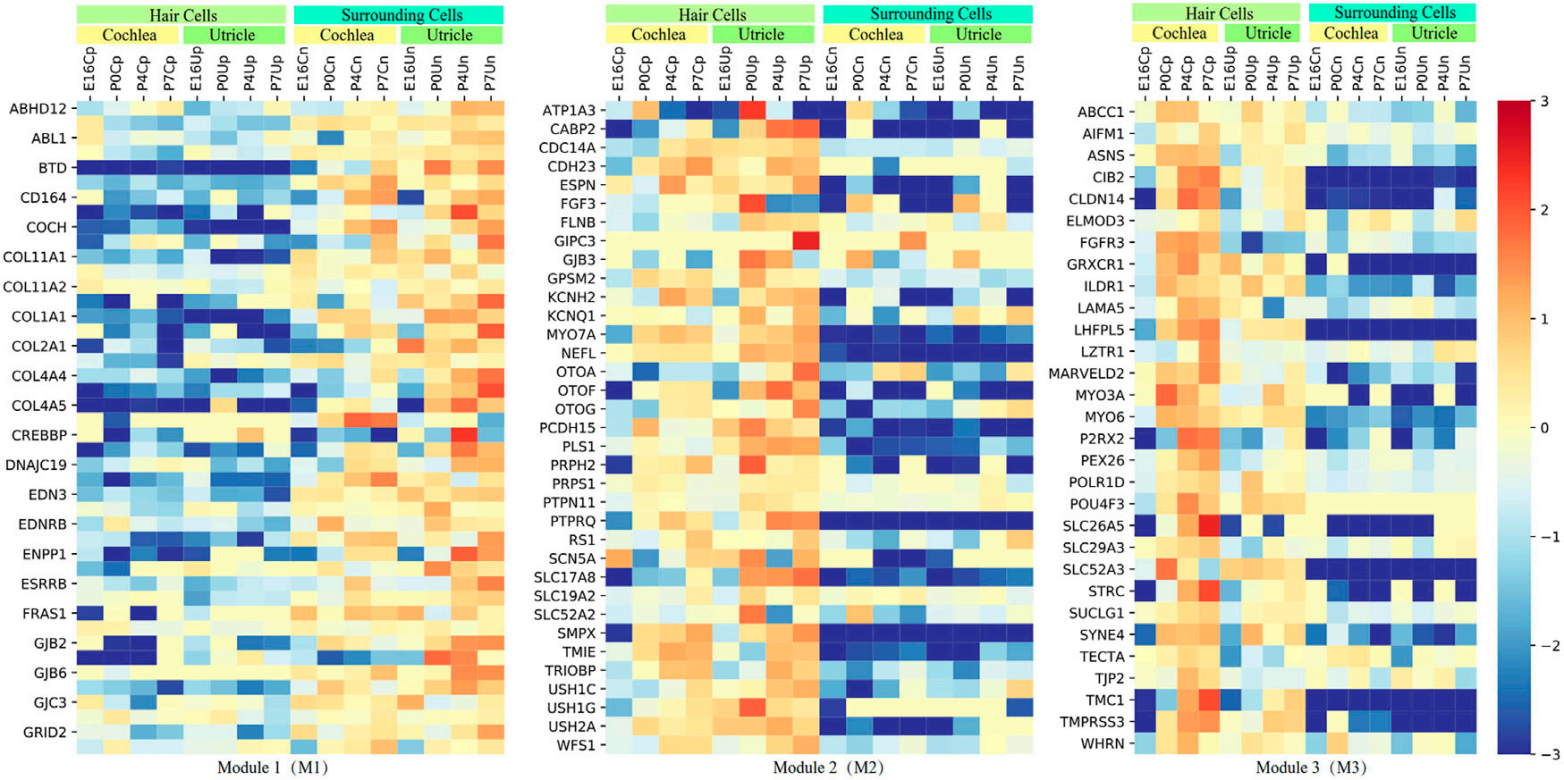
The spatiotemporal-expression patterns in the mouse inner-ear organs (cochlea and utricle) at four developmental stages. The three spatiotemporal expression patterns (M1, M2, or M3) for network genes are based on RNAseq data from FACS.

Next, we performed WGCNA in the tissue samples from FACS and identified three independent modules (M1-M3) comprising 109 genes to characterize the spatiotemporal-expression patterns of the 164 HL-related genes in convergent networks during inner-ear development (Figure 3 and Supplementary Table S3). Compared with the surrounding cells, we found that majority of genes within M1 (*n* = 44) were expressed lower in the hair cells of cochlea and utricle during E16, P0, P4, and P7 periods. In contrast, the expression of M2 genes (n = 35) was elevated in the hair cells of the cochlea and utricle. The pattern of gene expression in the surrounding cells of the cochlea and utricle within the M3 module was similar to the M2 module (n = 30). However, the gene expression in the hair cells of the cochlea and utricle within M2 was lower in the hair cells of utricle during the P0, P4, and P7 periods.

### HL-Related Genes Were Functionally Correlated

**FIGURE 4:**
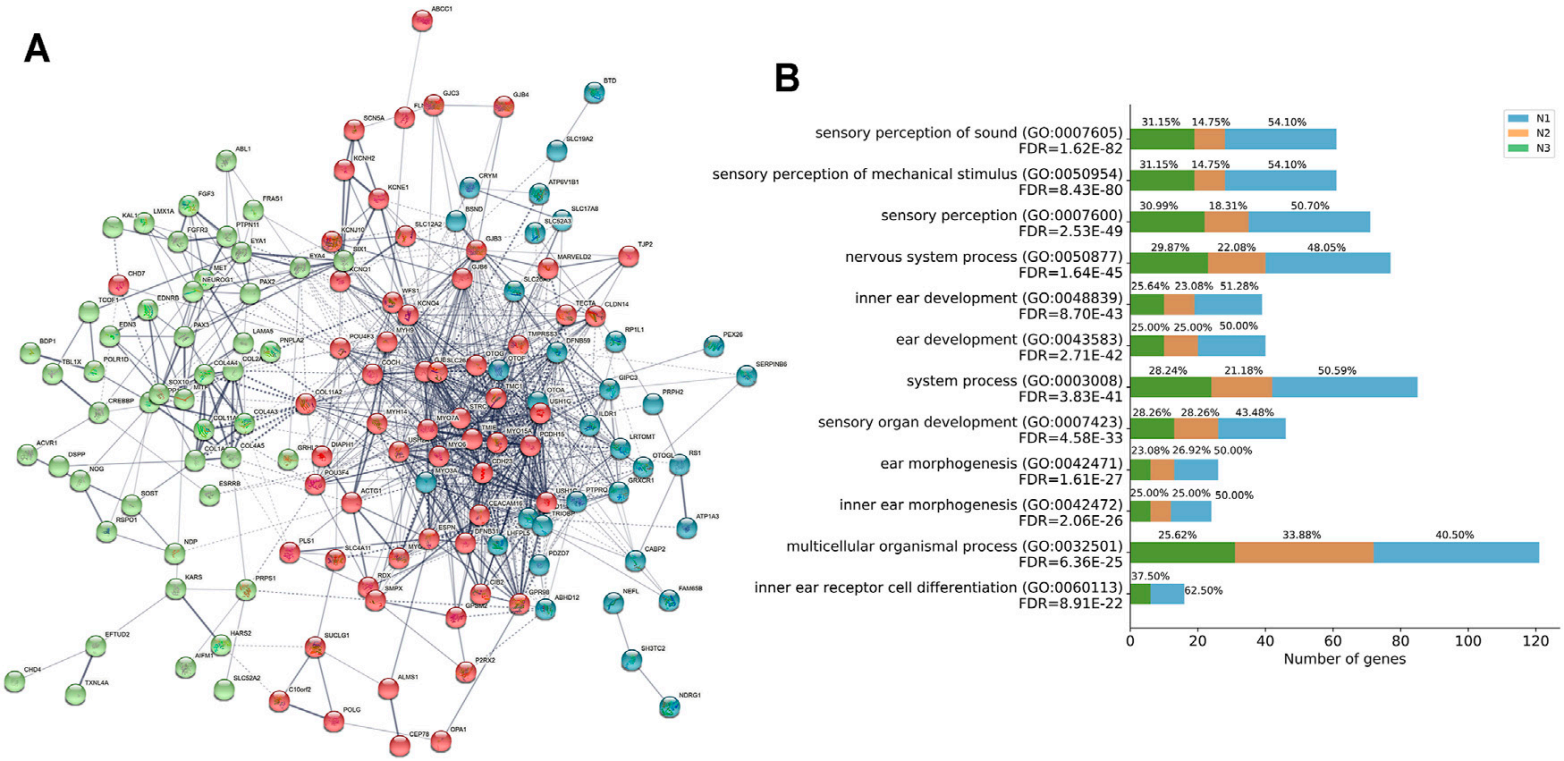
Functional network and biological progress of HL-related genes. **(A)** Functional network of closely related HL-related genes based on the STRING database. Three independent networks were identified by k-means clustering (N1 red, N2 green, and N3 blue). Nodes are colored to show the associations, and the thickness of lines connecting nodes indicates the strength of the association between nodes. **(B)** GO enrichment analysis for the contribution of genes in three independent networks N1, N2, and N3 to the functional signaling pathways.

Furthermore, we performed PPI base on the STRING v11.0 database and identified three independent networks (N1 red, N2 green, and N3 blue) to characterize the protein-protein interaction (PPI) patterns of the 164 genes in convergent networks. Specifically, the interacted functional network had 163 of the 164 HL-related genes with 930 connections of each other at protein-level (PPI enrichment *P*-value: < 1.0e-16) (Figure 4A and Supplementary Table S4).

GO enrichment analysis of the 163 genes identified several pathways associated with HL (Figure 4B and Supplementary Table S5). Most of these GO terms, such as sensory perception of sound (GO:0007605, FDR = 1.62E-82), sensory perception of mechanical stimulus (GO:0050954, FDR = 8.43E-80), sensory perception (GO:0007600, FDR = 2.53E-49), nervous system process (GO:0050877, FDR = 1.64E-45), inner ear development (GO:0048839, FDR = 8.70E-43), ear development (GO:0043583, FDR = 2.71E-42), system process (GO:0003008, FDR = 3.83E-41), sensory organ development (GO:0007423, FDR = 4.58E-33), ear morphogenesis (GO:0042471, FDR = 1.61E-27), inner ear morphogenesis (GO:0042472, FDR = 2.06E-26), multicellular organismal process (GO:0032501, FDR = 6.36E-25), inner ear receptor cell differentiation (GO:0060113, FDR = 8.91E-22), were regarded as critical functional signaling pathways associated with HL. The functional network suggested that the prioritized HL-related genes shared a common signaling mechanism and were functionally correlated.

In addition, we inspected the contribution of genes in three independent networks N1, N2, and N3, to the functional signaling pathways analyzed by GO enrichment analysis (Figure 4B). The contribution of the number of genes in N1 accounted for more than 50% for the following pathways: sensory perception of sound (GO:0007605), sensory perception of mechanical stimulus (GO:0050954), sensory perception (GO:0007600), inner ear development (GO:0048839), ear development (GO:0043583), system process (GO:0003008), ear morphogenesis (GO:0042471), inner ear morphogenesis (GO:0042472), inner ear receptor cell differentiation (GO:0060113).

## Discussion

The development of sequencing technologies has improved the rate of molecular diagnostic of HL, promoting our knowledge regarding the pathogenesis of hereditary HL with high clinical and genetic heterogeneity. The diagnostic rate of hereditary HL varies from 35 to 60% in different countries (Christina et al., 2016; Moteki et al., 2016; Rubén et al., 2018). Since 2003, our team has been working on the molecular etiologies and precaution of hereditary HL, and up to now, 22,456 cases have been tested for the identification of molecular etiologies. Various advancements have been made in the study on the pathogenic factors in the Chinese HL population (Dai et al., 2009a; Dai et al., 2009b; Dai et al., 2019; Yuan et al., 2020), resulting in the identification of new genes (Yuan et al., 2014; Gao et al., 2018a; Zhao et al., 2019) or novel variants (Gao et al., 2018b; Gao et al., 2020; Huang et al., 2020). We could identify the molecular etiology through gene sequencing in 52.19% of patients with HL (Yuan et al., 2020). Through the molecular epidemiological study on HL, a detailed mutational spectrum was revealed in the Chinese population with HL, and we found a high carrier rate (>15%) of common deafness gene variants in the normal hearing population. Thus, concurrent hearing and genetic screening in newborns were performed since 2012, and more than 10 million newborns in China benefited from the combined screening, which promoted the early detection and diagnosis of congenital deafness, which triggered intervention, prediction of late-onset and progressive hearing loss, and identification of individuals who were at risk of drug-induced HL (Dai et al., 2019). Also, a preimplantation genetic testing (PGT) procedure combining multiple annealing and looping-based amplification cycles (MALBAC) and single-nucleotide polymorphisms (SNPs) linkage analyses with a single low-depth next-generation sequencing run was done in 47 HL at-risk families with confirmed molecular diagnoses for HL to prevent the reoccurrence of HL (data not published). Additionally, genetic testing was helpful in predicting the language rehabilitation effect of HL patients who received cochlear implantation, a type of clinical treatment for severe to profound sensorineural HL. Although genetic diagnosis has extensive benefits to HL patients and high-risk families, the high heterogeneity of HL still creates some bottlenecks for the analysis of hereditary HL, such as the difficulties in judging variant pathogenicity and in interpreting the relationships between genotypes and phenotypes. Therefore, it is very important to build a comprehensive genetic resource database of HL, which can help in systematic integration of data from various studies and in obtaining useful biological information from the genetic discovery of HL.

As mentioned in the introduction section, although there are several public databases related to HL, Gene4HL has been designed as a one-stop search and analysis database of HL genes and variations, which offers significant advantages. First, the web interface of Gene4HL is easy to operate, which can provide an intuitive online interface for researchers to obtain more comprehensive genetic information regarding HL in a short time. Second, Gene4HL integrates almost all the gene-clinical phenotype and variation-clinical phenotype information, which are scattered in the published studies, which can provide more accurate genetic information. For example, c.109G > A (p.V37I) in *GJB2* was once controversial for its pathogenicity and is characteristic of the diversities of the HL phenotype it corresponds to. In Gene4HL, 146 articles about this variation were included. Here, all relevant details about *GJB2* c.109G > A have been displayed, including the basic information (in silico missense prediction, allele frequency in different populations, multiple Sequence Alignment retrieved from HomoloGene, and the disease-related information) and the detailed information from the original 146 articles, including the testing method, ethnic origin, total genetic testing samples, and the positive samples, and all the clinical phenotypes associated with the variation containing mild, moderate, severe to profound sensorineural hearing loss. Therefore, Gene4HL provides users with the relevant information of each variation comprehensively. In addition, Gene4HL also has an analytical function, which is not available in other public databases. Researchers can set genetic data at different standards according to their specific needs, analyze the original genetic data in this web interface, and flexibly prioritize candidate genes and variations. Next, we prioritized 164 HL-related genes, including 71 high-confidences, 42 strongly associated, and 52 suggestively associated genes. Of the known HL-causing genes, 59 belonged to the high-confidence genes, highlighting that the other 11 high-confidence HL-related genes were probably associated with HL. Therefore, additional genetic and experimental studies are needed to validate the genetic mechanisms of HL-related genes incorporating these factors. For example, *PTPN11*, the variations in *PTPN11* are the primary cause of Noonan syndrome with multiple lentigines and Noonan syndrome, which have common skin and facial symptoms, cardiac abnormalities and growth retardation, and hearing loss is considered to be a rare feature in these patients (Qiu et al., 1998; Niemeyer, 2014). However, in our previous cohort, we identified a group of patients with *PTPN11* pathogenic variants that were primarily manifested in congenital sensorineural hearing loss (Gao et al., 2020). *PTPN11* belongs to a high-confidence gene according to our classification; however, it has not been included in the known HL-related gene (Van et al., 2021). Therefore, through our classification of the included genes, Gene4HL could give users reminders of some HL-related genes that were previously not considered.

Furthermore, we used signed hybrid-weighted gene co-expression network analysis to characterize the spatiotemporal-expression patterns of genes of the mouse inner-ear organs (cochlea and utricle). The expression patterns of genes in the M1 and M2 clusters were similar to previous reports related to HL. For example, in the inner ear of mice and humans, the Cx26 protein encoded by *GJB2* (the most common HL-gene related to DFNB1A and DFNA3A) was not expressed in hair cells but expressed in different types of supporting cells in the cochlear epithelium (Liu et al., 2008; Azaiez et al., 2003). In mouse cochlea, Cx26 was first detected in a few cells on day 14.5 of the embryo, and the expression region of Cx26 gradually expanded during postnatal development (Sun et al., 2005). In humans, variants in cadherin 23 (*CDH23*) is known to cause Usher’s syndrome type D, as well as certain forms of NSHL, DFNB12. This gene expresses in the hair cells of human and mouse inner ears, and it is required for the proper organization of hair cell stereocilia. At embryonic day 18.5, the outer hair cells of Cdh23 homozygote mutant mice appear immature (Ralph et al., 2002). The expression profiles of genes in the M3 clusters were found to be similar to M2 except that the gene expression in the hair cells of cochlea and utricle within M2 were lower in the hair cells of utricle during P0, P4, and P7 periods. Another example includes *OTOF*, which is related to DFNB9 and encodes otoferlin, a large transmembrane vesicular Ca^2+^ binding protein with six C2 domains, which operates as the main Ca^2+^ sensor for neurotransmitter release at inner hair cells (IHCs) ribbon synapses and type I vestibular hair cells and immature outer hair cells (OHCs). In the absence of otoferlin, signal transmission of IHCs fails due to the impaired release of synaptic vesicles at the IHC synapse (Dulon et al., 2009). Therefore, this method could be easily used to characterize spatiotemporal-expression patterns of genes.

Moreover, we performed analyses to validate the functional association of the 164 HL-related genes based on PPI networks. We observed significant associations among the HL-related genes, which indicated that 163 of 164 HL-related genes interacted with each other. We also performed PPI and co-expression analyses to investigate the related functional pathways, with results converging on sensory perception of sound, sensory perception of the mechanical stimulus, sensory perception, inner ear development, ear development, system process, ear morphogenesis, inner ear morphogenesis, and inner ear receptor cell differentiation. These results suggested that those 163 genes were involved in auditory biological functions, and defects of them increased the risk of HL.

However, this study has several limitations. First, the integrated approach in the present study might deviate, and the prioritized genes need validation in different populations and verification of their pathogenic mechanisms by cellular or animal experiments. If there is conflicting predictions, we think, under most circumstances, results from functional experiments may be more reliable compared to that produced by in silico prediction. Second, hearing loss is a kind of disease with high heterogeneity and the gene prioritization scoring method doesn’t provide clear path of how it should be incorporated on a case-by-case basis in the context of the heterogeneity. Third, we did not include noncoding variants and copy number variants (CNVs) in this study. Since noncoding variants and CNVs also play an important role in HL, we will focus on them in the next phase. In this regard, we encourage researchers to provide their own data and contact us to refine the missing data of HL. We will keep continuously updating the Gene4HL database annually. Lastly, functional mechanisms of genes in their impact on the hearing loss levels had not been taken into account when classifying the genes.

Thus, we cataloged different types of genetic data from 1,608 publications related to HL and prioritized 164 HL-related genes, which were used to construct the database and analysis tool Gene4HL. Moreover, we describe the genetic landscape of prioritized HL-related genes, providing insight into HL pathology. Thus, Gene4HL provides comprehensive genetic knowledge and analytic platform of HL for researchers and clinicians, accelerating the understanding of pathogenesis of HL.

## Data Availability

The datasets presented in this study can be found in online repositories. The names of the repository/repositories and accession number(s) can be found in the article/Supplementary Material.
