# Supplementary figures and images for: Gene4HL: An Integrated Genetic Database for Hearing Loss
Source: Front Genet. 2021 Oct 18;12:773009. doi: 10.3389/fgene.2021.773009 (PMC8558372; doi:10.3389/fgene.2021.773009)

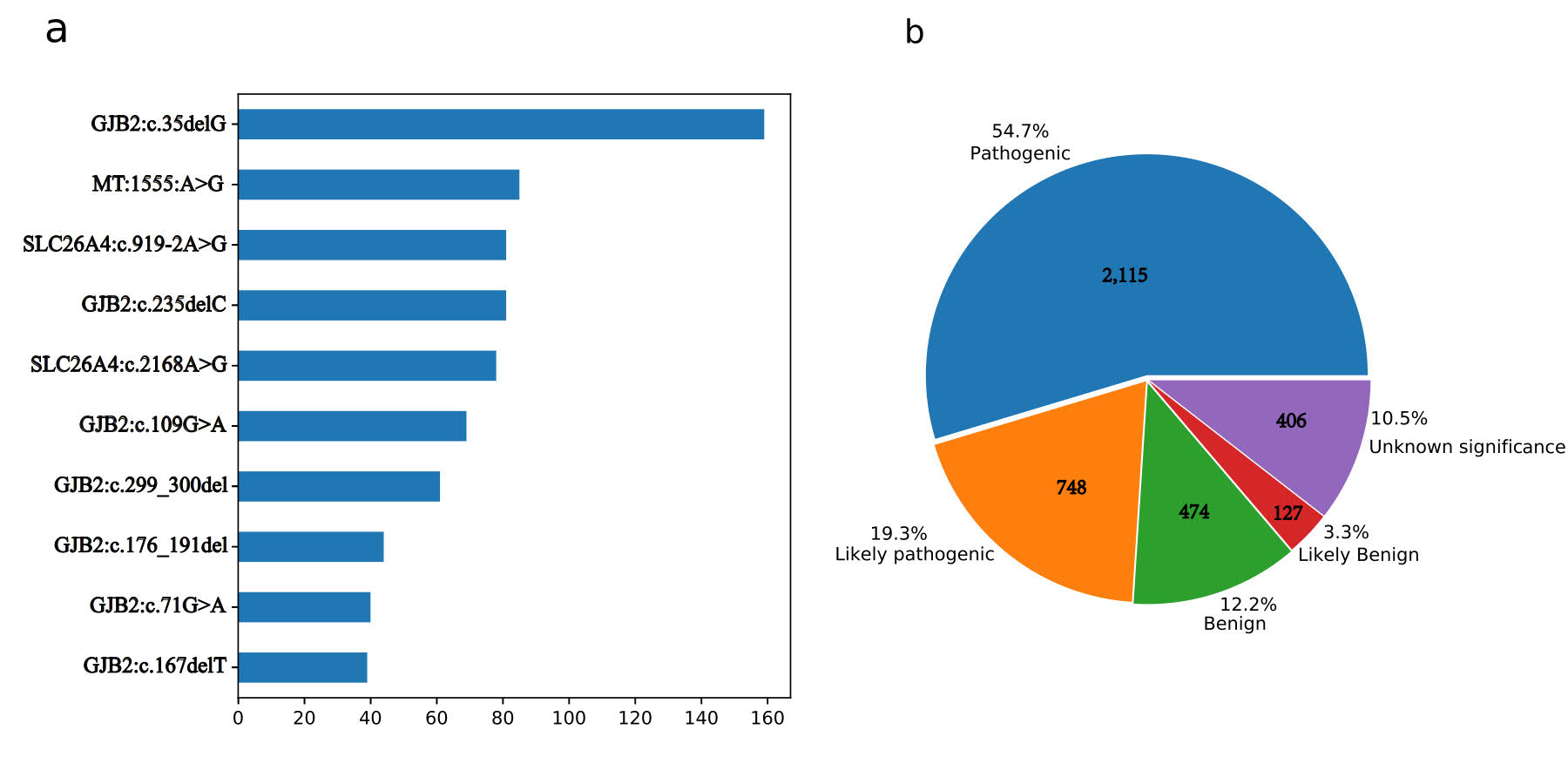

Supplement: Supplementary file 2 [file Image1.JPEG]

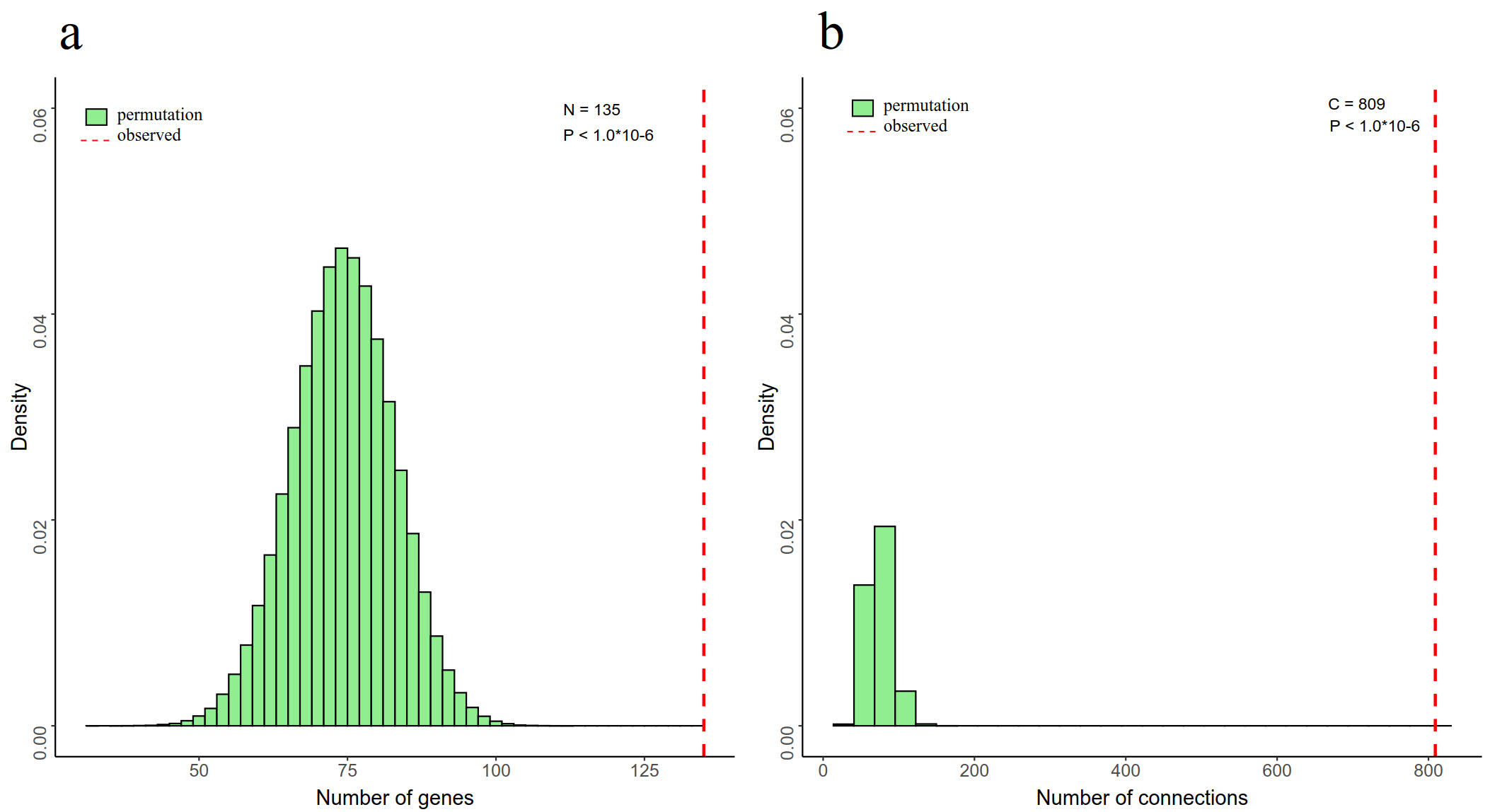

Supplement: Supplementary file 3 [file Image2.JPEG]
