# Supplementary material for: Gene4HL: An Integrated Genetic Database for Hearing Loss
Source: Front Genet. 2021 Oct 18;12:773009. doi: 10.3389/fgene.2021.773009 (PMC8558372; doi:10.3389/fgene.2021.773009)
Supplement: Supplementary file 4 [file Table2.DOCX]

Table S2. Weight scheme of various genetic data for prioritizing candidate genes.

| **Variants^*^** | **Score value** |
| --- | --- |
| loss-of-function variants**^a^** | **5** |
| damaging variants**^b^** | **3** |
| tolerate misense**^c^** | **2** |
| synonymous**^d^** | **1** |
| nonframeshift**^e^** | **1** |
| remaining variants | **1** |

*. Annotation by Annovar and ReVe.

1. loss-of-function: stopgain, stoploss, splicing
2. damaging missense: ReVe > 0.7
3. tolerate misense: ReVe <= 0.7
4. synonymous: synonymous
5. nonframeshift: nonframeshift deletion, nonframeshift insertion, nonframeshift substitution
