# Supplementary material for: Gene4HL: An Integrated Genetic Database for Hearing Loss
Source: Front Genet. 2021 Oct 18;12:773009. doi: 10.3389/fgene.2021.773009 (PMC8558372; doi:10.3389/fgene.2021.773009)
Supplement: Supplementary file 5 [file Table5.DOCX]

Table S5. Three independent networks (N1 red, N2 green and N3 blue) are identified by kmeans clustering.

| **#clustering method** | **cluster number** | **cluster color** | **protein name** |
| --- | --- | --- | --- |
| kmeans | 1 | Red | ABCC1 |
| kmeans | 1 | Red | ACTG1 |
| kmeans | 1 | Red | ALMS1 |
| kmeans | 1 | Red | C10orf2 |
| kmeans | 1 | Red | CDH23 |
| kmeans | 1 | Red | CEACAM16 |
| kmeans | 1 | Red | CEP78 |
| kmeans | 1 | Red | CHD7 |
| kmeans | 1 | Red | CIB2 |
| kmeans | 1 | Red | CLDN14 |
| kmeans | 1 | Red | COCH |
| kmeans | 1 | Red | COL11A2 |
| kmeans | 1 | Red | DFNB31 |
| kmeans | 1 | Red | DIAPH1 |
| kmeans | 1 | Red | ESPN |
| kmeans | 1 | Red | FLNB |
| kmeans | 1 | Red | GJB2 |
| kmeans | 1 | Red | GJB3 |
| kmeans | 1 | Red | GJB4 |
| kmeans | 1 | Red | GJB6 |
| kmeans | 1 | Red | GJC3 |
| kmeans | 1 | Red | GPR98 |
| kmeans | 1 | Red | GPSM2 |
| kmeans | 1 | Red | KCNE1 |
| kmeans | 1 | Red | KCNH2 |
| kmeans | 1 | Red | KCNJ10 |
| kmeans | 1 | Red | KCNQ1 |
| kmeans | 1 | Red | KCNQ4 |
| kmeans | 1 | Red | MARVELD2 |
| kmeans | 1 | Red | MYH14 |
| kmeans | 1 | Red | MYH9 |
| kmeans | 1 | Red | MYO15A |
| kmeans | 1 | Red | MYO1A |
| kmeans | 1 | Red | MYO6 |
| kmeans | 1 | Red | MYO7A |
| kmeans | 1 | Red | OPA1 |
| kmeans | 1 | Red | OTOG |
| kmeans | 1 | Red | P2RX2 |
| kmeans | 1 | Red | PCDH15 |
| kmeans | 1 | Red | PLS1 |
| kmeans | 1 | Red | POLG |
| kmeans | 1 | Red | POU3F4 |
| kmeans | 1 | Red | POU4F3 |
| kmeans | 1 | Red | RDX |
| kmeans | 1 | Red | SCN5A |
| kmeans | 1 | Red | SLC12A2 |
| kmeans | 1 | Red | SLC26A4 |
| kmeans | 1 | Red | SLC4A11 |
| kmeans | 1 | Red | SMPX |
| kmeans | 1 | Red | STRC |
| kmeans | 1 | Red | SUCLG1 |
| kmeans | 1 | Red | TECTA |
| kmeans | 1 | Red | TJP2 |
| kmeans | 1 | Red | TMC1 |
| kmeans | 1 | Red | TMIE |
| kmeans | 1 | Red | TMPRSS3 |
| kmeans | 1 | Red | USH1C |
| kmeans | 1 | Red | USH1G |
| kmeans | 1 | Red | USH2A |
| kmeans | 1 | Red | WFS1 |
| kmeans | 2 | Green | ABL1 |
| kmeans | 2 | Green | ACVR1 |
| kmeans | 2 | Green | AIFM1 |
| kmeans | 2 | Green | ANKRD11 |
| kmeans | 2 | Green | BDP1 |
| kmeans | 2 | Green | CHD4 |
| kmeans | 2 | Green | COL11A1 |
| kmeans | 2 | Green | COL1A1 |
| kmeans | 2 | Green | COL2A1 |
| kmeans | 2 | Green | COL4A3 |
| kmeans | 2 | Green | COL4A4 |
| kmeans | 2 | Green | COL4A5 |
| kmeans | 2 | Green | CREBBP |
| kmeans | 2 | Green | DNAJC19 |
| kmeans | 2 | Green | DSPP |
| kmeans | 2 | Green | EDN3 |
| kmeans | 2 | Green | EDNRB |
| kmeans | 2 | Green | EFTUD2 |
| kmeans | 2 | Green | ENPP1 |
| kmeans | 2 | Green | ERCC8 |
| kmeans | 2 | Green | ESRRB |
| kmeans | 2 | Green | EYA1 |
| kmeans | 2 | Green | EYA4 |
| kmeans | 2 | Green | FGF3 |
| kmeans | 2 | Green | FGFR3 |
| kmeans | 2 | Green | FRAS1 |
| kmeans | 2 | Green | GRHL2 |
| kmeans | 2 | Green | GRID2 |
| kmeans | 2 | Green | HARS2 |
| kmeans | 2 | Green | KAL1 |
| kmeans | 2 | Green | KARS |
| kmeans | 2 | Green | LAMA5 |
| kmeans | 2 | Green | LMX1A |
| kmeans | 2 | Green | MAN2B1 |
| kmeans | 2 | Green | MET |
| kmeans | 2 | Green | MITF |
| kmeans | 2 | Green | NDP |
| kmeans | 2 | Green | NEUROG1 |
| kmeans | 2 | Green | NOG |
| kmeans | 2 | Green | PAX2 |
| kmeans | 2 | Green | PAX3 |
| kmeans | 2 | Green | PNPLA2 |
| kmeans | 2 | Green | POLR1D |
| kmeans | 2 | Green | PRPS1 |
| kmeans | 2 | Green | PTPN11 |
| kmeans | 2 | Green | RSPO1 |
| kmeans | 2 | Green | SIX1 |
| kmeans | 2 | Green | SLC29A3 |
| kmeans | 2 | Green | SLC52A2 |
| kmeans | 2 | Green | SOST |
| kmeans | 2 | Green | SOX10 |
| kmeans | 2 | Green | TBC1D24 |
| kmeans | 2 | Green | TBL1X |
| kmeans | 2 | Green | TCOF1 |
| kmeans | 2 | Green | TXNL4A |
| kmeans | 2 | Green | ZBTB20 |
| kmeans | 3 | Blue | ABHD12 |
| kmeans | 3 | Blue | ALG11 |
| kmeans | 3 | Blue | ASNS |
| kmeans | 3 | Blue | ATP1A3 |
| kmeans | 3 | Blue | ATP6V1B1 |
| kmeans | 3 | Blue | BSND |
| kmeans | 3 | Blue | BTD |
| kmeans | 3 | Blue | CABP2 |
| kmeans | 3 | Blue | CD164 |
| kmeans | 3 | Blue | CDC14A |
| kmeans | 3 | Blue | CRYM |
| kmeans | 3 | Blue | DFNB59 |
| kmeans | 3 | Blue | ELMOD3 |
| kmeans | 3 | Blue | FAM136A |
| kmeans | 3 | Blue | FAM65B |
| kmeans | 3 | Blue | GIPC3 |
| kmeans | 3 | Blue | GRXCR1 |
| kmeans | 3 | Blue | ILDR1 |
| kmeans | 3 | Blue | KCNE1B |
| kmeans | 3 | Blue | LHFPL5 |
| kmeans | 3 | Blue | LOXHD1 |
| kmeans | 3 | Blue | LRTOMT |
| kmeans | 3 | Blue | LZTR1 |
| kmeans | 3 | Blue | MSL3 |
| kmeans | 3 | Blue | MYO3A |
| kmeans | 3 | Blue | NDRG1 |
| kmeans | 3 | Blue | NEFL |
| kmeans | 3 | Blue | NLRP3 |
| kmeans | 3 | Blue | OTOA |
| kmeans | 3 | Blue | OTOF |
| kmeans | 3 | Blue | OTOGL |
| kmeans | 3 | Blue | PDZD7 |
| kmeans | 3 | Blue | PEX26 |
| kmeans | 3 | Blue | PRPH2 |
| kmeans | 3 | Blue | PTPRQ |
| kmeans | 3 | Blue | RMND1 |
| kmeans | 3 | Blue | RP1L1 |
| kmeans | 3 | Blue | RS1 |
| kmeans | 3 | Blue | SERPINB6 |
| kmeans | 3 | Blue | SH3TC2 |
| kmeans | 3 | Blue | SLC17A8 |
| kmeans | 3 | Blue | SLC19A2 |
| kmeans | 3 | Blue | SLC26A5 |
| kmeans | 3 | Blue | SLC52A3 |
| kmeans | 3 | Blue | SYNE4 |
| kmeans | 3 | Blue | TRIOBP |
| kmeans | 3 | Blue | XYLT2 |
